# Effects of Dapagliflozin in Chronic Kidney Disease, With and Without Other Cardiovascular Medications: DAPA‐CKD Trial

**DOI:** 10.1161/JAHA.122.028739

**Published:** 2023-04-29

**Authors:** Glenn M. Chertow, Ricardo Correa‐Rotter, Priya Vart, Niels Jongs, John J. V. McMurray, Peter Rossing, Anna Maria Langkilde, C. David Sjöström, Robert D. Toto, David C. Wheeler, Hiddo J. L. Heerspink, for the DAPA‐CKD Trial Committees and Investigators

**Affiliations:** ^1^ Departments of Medicine, Epidemiology and Population Health, and Health Policy Stanford University School of Medicine Stanford CA; ^2^ Instituto Nacional de Ciencias Médicas y Nutrición Salvador Zubirán Mexico City Mexico; ^3^ Department of Clinical Pharmacy and Pharmacology University of Groningen, University Medical Centre Groningen Groningen The Netherlands; ^4^ Institute of Cardiovascular and Medical Sciences University of Glasgow Glasgow UK; ^5^ Steno Diabetes Center Copenhagen Herlev Denmark; ^6^ Department of Clinical Medicine University of Copenhagen Copenhagen Denmark; ^7^ Late‐Stage Development, Cardiovascular, Renal and Metabolism, BioPharmaceuticals R&D AstraZeneca Gothenburg Sweden; ^8^ Department of Internal Medicine UT Southwestern Medical Center Dallas TX; ^9^ Department of Renal Medicine University College London London UK; ^10^ The George Institute for Global Health Sydney Australia

**Keywords:** cardiovascular medications, chronic kidney disease, dapagliflozin, SGLT2 inhibitors, Treatment, Clinical Studies, ACE/Angiotension Receptors/Renin Angiotensin System

## Abstract

**Background:**

The DAPA‐CKD (Dapagliflozin and Prevention of Adverse Outcomes in Chronic Kidney Disease) trial (NCT03036150) demonstrated that dapagliflozin reduced the risk of kidney and cardiovascular events in patients with chronic kidney disease and albuminuria with and without type 2 diabetes. We aimed to determine whether baseline cardiovascular medication use modified the dapagliflozin treatment effect.

**Methods and Results:**

We randomized 4304 adults with baseline estimated glomerular filtration rate 25 to 75 mL/min per 1.73 m^2^ and urinary albumin:creatinine ratio 200 to 5000 mg/g to dapagliflozin 10 mg or placebo once daily. The primary end point was a composite of ≥50% estimated glomerular filtration rate decline, end‐stage kidney disease, and kidney or cardiovascular death. Secondary end points included a kidney composite end point (primary composite end point without cardiovascular death), a cardiovascular composite end point (hospitalized heart failure or cardiovascular death), and all‐cause mortality. We categorized patients according to baseline cardiovascular medication use/nonuse. Patients were required by protocol to receive a stable (and maximally tolerated) dose of a renin‐angiotensin‐aldosterone system inhibitor. We observed consistent benefits of dapagliflozin relative to placebo, irrespective of baseline use/nonuse of renin‐angiotensin‐aldosterone system inhibitors (98.1%), calcium channel blockers (50.7%), β‐adrenergic antagonists (39.0%), diuretics (43.7%), and antithrombotic (47.4%), and lipid‐lowering (15.0%) agents. Use of these drugs in combination with dapagliflozin did not increase the number of serious adverse events.

**Conclusions:**

The safety profile and efficacy of dapagliflozin on kidney and cardiovascular end points in patients with chronic kidney disease were consistent among patients treated and not treated at baseline with a range of cardiovascular medications.

**Registration Information:**

clinicaltrials.gov. Identifier: NCT03036150.

**Nonstandard Abbreviations and Acronyms**
AE: adverse event
CANVAS: Canagliflozin Cardiovascular Assessment Study
CKD‐EPI: Chronic Kidney Disease Epidemiology Collaboration
CREDENCE: Canagliflozin and Renal Events in Diabetes with Established Nephropathy Clinical Evaluation
CRT: cardiac resynchronization therapy
DAPA‐CKD: Dapagliflozin and Prevention of Adverse Outcomes in Chronic Kidney Disease
DECLARE‐TIMI 58: Dapagliflozin Effect on Cardiovascular Events–Thrombolysis in Myocardial Infarction 58
EMPA‐REG: (Empagliflozin) Cardiovascular Outcome
EMPEROR‐Reduced: Empagliflozin Outcome Trial in Patients With Chronic Heart Failure With Reduced Ejection Fraction
FIDELIO‐DKD: Finerenone in Reducing Kidney Failure and Disease Progression in Diabetic Kidney Disease
NC: non calculable
RAAS: renin‐angiotensin‐aldosterone system
SGLT2: sodium‐glucose cotransporter‐2

**Clinical Perspective**

**What Is New?**

- The DAPA‐CKD (Dapagliflozin and Prevention of Adverse Outcomes in Chronic Kidney Disease) trial demonstrated that compared with placebo, dapagliflozin reduced adverse kidney and cardiovascular outcomes and all‐cause mortality in patients with chronic kidney disease, irrespective of the presence of diabetes; however, it is unknown whether the use of cardiovascular medications modifies these effects of dapagliflozin in this patient population.
- We investigated the benefits of dapagliflozin in participants treated and not treated with a range of cardiovascular medications—diuretics, calcium channel blockers, β‐adrenergic antagonists, antithrombotic agents, and lipid‐lowering agents—used as standard of care in the aforementioned patient population; results showed that the efficacy of dapagliflozin on kidney and cardiovascular end points was consistent, with no change in associated safety, irrespective of treatment with a range of cardiovascular medications.

**What Are the Clinical Implications?**

- These results document the safety and efficacy of dapagliflozin with use/nonuse of a wide range of cardiovascular medications, demonstrating benefits on kidney and cardiovascular outcomes in patients with mild, moderate, and advanced chronic kidney disease, with and without diabetes, which are populations at higher risk of adverse drug effects relative to persons with normal or near normal kidney function. Furthermore, the demonstrated safety of coadministration of dapagliflozin with a range of antithrombotic and lipid‐lowering agents may allow patients to enjoy the benefits of dapagliflozin on reducing risks of heart failure and progressive kidney disease, while safely gaining treatment benefits with other agents aimed to reduce cardiovascular complications, thus expanding the treatment repertoire for this patient population.

In the DAPA‐CKD (Dapagliflozin and Prevention of Adverse outcomes in Chronic Kidney Disease)) trial, the sodium‐glucose cotransporter‐2 (SGLT2) inhibitor dapagliflozin reduced the risk of major kidney and cardiovascular events and prolonged survival in patients with chronic kidney disease (CKD), with and without type 2 diabetes.^1^ Similar results had been observed in patients with type 2 diabetes in the CREDENCE (Canagliflozin and Renal Events in Diabetes with Established Nephropathy Clinical Evaluation) trial, in which canagliflozin significantly reduced the risks of progressive kidney disease and major cardiovascular events but not cardiovascular mortality.^2^ Previous studies with these and other SGLT2 inhibitors in patients with type 2 diabetes demonstrated significant cardiovascular benefits (EMPA‐REG (Empagliflozin) Cardiovascular Outcome), DECLARE‐TIMI 58 (Dapagliflozin Effect on Cardiovascular Events–Thrombolysis in Myocardial Infarction 58), and CANVAS (Canagliflozin Cardiovascular Assessment Study).^2^, ^3^, ^4^

Patients included in these and other cardiovascular trials are typically treated with a variety of concomitant medications, many of which are indicated either for control of hypertension or for prevention or treatment of other cardiovascular conditions (eg, hypercholesterolemia, coronary, cerebrovascular or peripheral arterial disease, heart failure, and atrial fibrillation). In DAPA‐CKD and other trials, maximally tolerated doses of renin‐angiotensin‐aldosterone system (RAAS) inhibitors were required by protocol.

The DAPA‐CKD trial required that all patients be treated with standard of care (regional guideline–concordant) therapies for kidney, cardiovascular, and other conditions unless contraindicated.^1^ The protocol itself did not mandate specific drugs or doses, with the exception of the RAAS inhibitors, as previously described.

In this prespecified analysis of the DAPA‐CKD trial, we aimed to examine whether baseline use of cardiovascular medications modified the effects of dapagliflozin on kidney and cardiovascular end points and on safety in patients with CKD and albuminuria.

## Methods

### Data Sharing Statement

Data underlying the findings described in this article may be obtained in accordance with AstraZeneca's data sharing policy described at https://astrazenecagrouptrials.pharmacm.com/ST/Submission/Disclosure.

Data for studies directly listed on Vivli can be requested through Vivli at www.vivli.org. Data for studies not listed on Vivli can be requested through Vivli at https://vivli.org/members/enquiries‐about‐studies‐not‐listed‐on‐the‐vivli‐platform/. AstraZeneca Vivli member page is also available outlining further details: https://vivli.org/ourmember/astrazeneca/.

DAPA‐CKD was a randomized, double‐blind, placebo‐controlled, multicenter clinical trial; articles describing trial design, baseline characteristics, primary results, and results stratified by diabetes status, history of cardiovascular disease, and several other baseline clinical characteristics have been previously published.^1^, ^5^ The trial was sponsored by AstraZeneca and conducted at 386 sites in 21 countries from February 2017 through June 2020 and registered at clinicaltrials.gov (NCT03036150). The safety of participants in the trial was overseen by an independent Data and Safety Monitoring Committee. The DAPA‐CKD trial was conducted according to the principles of the Declaration of Helsinki. Ethics committees at all participating centers approved the protocol, and all participants provided written informed consent before any study‐specific procedure commenced.

#### Participants

Adults with or without type 2 diabetes, estimated glomerular filtration rate (eGFR) 25 to 75 mL/min per 1.73 m^2^, and urinary albumin:creatinine ratio 200 to 5000 mg/g were eligible for participation. We required patients to be treated with a stable maximally tolerated dose of RAAS inhibitor (angiotensin‐converting enzyme [ACE] inhibitor or angiotensin receptor blocker [ARB]) for ≥4 weeks, unless medically contraindicated. Key exclusion criteria included documented diagnosis of type 1 diabetes, polycystic kidney disease, lupus nephritis, or antineutrophil cytoplasmic antibody‐associated vasculitis. A complete list of inclusion and exclusion criteria and the trial protocol have been previously published.^1^

#### Procedures

Participants were randomly assigned to dapagliflozin 10 mg once daily or matching placebo, in accordance with the sequestered, fixed‐randomization schedule, with the use of balanced blocks to ensure an approximate 1:1 ratio of the 2 regimens. Randomization was stratified by diabetes status and urinary albumin:creatinine ratio (≤or >1000 mg/g). We calculated eGFR using the Chronic Kidney Disease Epidemiology Collaboration and incorporated results from the equation as originally defined, including a term for self‐reported race (Black versus non‐Black race). Recruitment of patients with eGFR 60 to 75 mL/min per 1.73 m^2^ was limited to no more than 10% of trial participants. Participants and all study personnel (except the Independent Data Monitoring Committee) were masked to treatment allocation.

After randomization, in‐person study visits were performed after 2 weeks, 2, 4, and 8 months, and at 4‐month intervals thereafter. At each follow‐up visit, study personnel recorded vital signs, obtained blood and urine samples, and recorded information on potential study end points, adverse events, concomitant therapies, and study drug adherence.

#### End Points

The primary composite end point was time to ≥50% decline in eGFR (confirmed by a second serum creatinine measurement after at least 28 days), onset of kidney failure (defined as maintenance dialysis for at least 28 days, kidney transplantation, or eGFR <15 mL/min per 1.73 m^2^ confirmed by a second measurement after at least 28 days), or death from a kidney or cardiovascular cause. Secondary end points were time to (1) a composite kidney end point of ≥50% sustained decline in eGFR, kidney failure, or death from kidney disease; (2) a composite cardiovascular end point defined as hospitalization for heart failure or cardiovascular death; and (3) death from any cause. We also prespecified change in eGFR slope as an exploratory efficacy end point. All efficacy end points were adjudicated by a masked, independent Clinical Events Committee, except for the quantitative assessments of eGFR, which were obtained from our central laboratory.

#### Cardiovascular Medication Use

Participants were required to receive the highest tolerated dose of RAAS inhibitors; all other agents were prescribed at the discretion of the treating physician in accordance with regional standards of care for associated conditions. We categorized cardiovascular medications as follows: diuretics (loop diuretic, thiazide diuretic, or mineralocorticoid receptor antagonist), calcium channel blockers, β‐adrenergic antagonists, antithrombotic agents (antiplatelet drugs or anticoagulants), and lipid‐lowering agents (statins or other). We further categorized patients according to baseline RAAS inhibitor dose, defined as 100% (or more), 50 to <100%, and <50% of the maximum labeled antihypertensive dose, and those not treated with RAAS inhibitors.

#### Statistical Analysis

The overall analytic approach, power calculation, and prespecified statistical analysis plan have been previously published.^1^ All analyses presented here followed the intention‐to‐treat principle. Briefly, we conducted time‐to‐event analyses using a proportional hazards (Cox) regression stratified by randomization factors (diabetes status and urinary albumin:creatinine ratio), adjusting for baseline eGFR, yielding hazard ratios and 95% CI from model parameter coefficients and SEs. For the purpose of the current analysis, we evaluated the primary and secondary efficacy end points in participants stratified by baseline use/nonuse of cardiovascular medication class. We tested for heterogeneity of the dapagliflozin treatment effect by including a multiplicative interaction term between randomized treatment group and use/nonuse of cardiovascular medications. For time‐to‐event analyses, we assessed for nonuniformity of hazard ratios with the Akaike's information criterion. We considered 2‐tailed *P* values <0.05 to be statistically significant.

## Results

As previously described,^1^ 4304 adults with and without type 2 diabetes with baseline eGFR 25 to 75 mL/min per 1.73 m^2^ and urinary albumin:creatinine ratio of 200 to 5000 mg/g were randomized to either dapagliflozin 10 mg or placebo once daily with a median follow‐up of 2.4 years. Table 1 shows selected demographic and clinical characteristics of participants in each of the randomized groups (dapagliflozin versus placebo) stratified by diabetes status, including baseline use/nonuse of cardiovascular medications.

**Table 1:** Baseline Characteristics of Participants Stratified by Diabetes Status at Baseline (All Randomized Participants)

Characteristic	With type 2 diabetes		Without type 2 diabetes	
	Dapagliflozin (n=1455)	Placebo (n=1451)	Dapagliflozin (n=697)	Placebo (n=701)
Age,y, mean (SD)	64.1 (9.8)	64.7 (9.5)	56.9 (14.6)	56.0 (14.6)
Female sex, n (%)	494 (34.0)	471 (32.5)	215 (30.8)	245 (35.0)
Weight,kg, mean (SD)	83.2 (20.9)	83.8 (21.2)	77.9 (17.8)	78.3 (19.9)
BMI,kg/m^2^, mean (SD)	30.2 (6.2)	30.4 (6.3)	27.7 (5.2)	28.1 (5.9)
Blood pressure,mm Hg, mean (SD)				
Systolic	138.8 (17.6)	139.6 (17.1)	132.3 (16.4)	132.9 (16.9)
Diastolic	76.5 (10.4)	76.5 (9.9)	79.6 (10.9)	79.6 (10.8)
eGFR,mL/min/1.73 m^2^	44.0 (12.6)	43.6 (12.6)	41.7 (11.5)	41.8 (11.9)
UACR,mg/g, median (IQR)	1024.5 (472.5–2111.0)	1004.5 (493.2–2017.0)	870.5 (472.0–1533.5)	841.5 (458.5–1554.5)
HbA1c,%, mean (SD)	7.8 (1.7)	7.8 (1.6)	5.6 (0.4)	5.6 (0.4)
History of cardiovascular disease, n (%)*	640 (44.0)	641 (44.2)	173 (24.8)	156 (22.2)
Prior cardiovascular medication, n (%)				
ACE inhibitors	451 (31.0)	443 (30.5)	222 (31.9)	238 (34.0)
ARB	984 (67.6)	974 (67.1)	460 (66.0)	452 (64.5)
Calcium channel blockers	767 (52.7)	782 (53.9)	307 (44.0)	327 (46.6)
Dihydropyridine†	738 (96.2)	759 (97.1)	293 (95.4)	313 (95.7)
Nondihydropyridine‡	29 (3.8)	23 (2.9)	14 (4.6)	14 (4.3)
β‐blockers	627 (43.1)	640 (44.1)	219 (31.4)	194 (27.7)
Diuretics	718 (49.3)	747 (51.5)	210 (30.1)	207 (29.5)
MRA	82 (5.6)	89 (6.1)	27 (3.9)	31 (4.4)
Antithrombotic agents§	820 (56.4)	829 (57.1)	202 (29.0)	191 (27.2)
Lipid‐lowering agents	209 (14.4)	243 (16.7)	111 (15.9)	82 (11.7)

ACE indicates angiotensin‐converting enzyme; ARB, angiotensin receptor blocker; BMI, body mass index; eGFR, estimated glomerular filtration rate; HbA1c, hemoglobin A1c; IQR, interquartile range; MRA, mineralocorticoid receptor antagonist; and UACR, urine albumin:creatinine ratio.

* Coronary heart disease, cerebrovascular disease, peripheral artery disease, heart failure, valvular heart disease, atrial fibrillation or atrial flutter, ventricular arrhythmia, pulmonary embolism, and cardiac devices other than those used for cardiac resynchronization therapy.

^†^ Includes amlodipine, nifedipine, isradipine, felodipine, nicardipine, and nisoldipine.

^‡^ Includes diltiazem and verapamil.

^§^ Antiplatelet agents and anticoagulants.

Cardiovascular medications were generally used more frequently in patients with diabetes, but there were no material differences in the proportion of patients using cardiovascular medications by randomized group. Of the 4304 randomized participants, 4224 (98.1%) were receiving a RAAS inhibitor at baseline, with roughly twice as many patients treated with ARBs as compared with ACE inhibitors. Among the 4296 (99.9%) participants with available data on baseline RAAS inhibitor dose, 1231 (28.6%), 1867 (43.5%), and 1068 (24.8%) were prescribed 100% (or more), 50 to <100%, and <50% of the maximum labeled antihypertensive dose, respectively, and 130 (3.0%) were not prescribed RAAS inhibitors.

### Efficacy of Dapagliflozin by Baseline Use/Nonuse of Cardiovascular Medications

**Figure 1:**
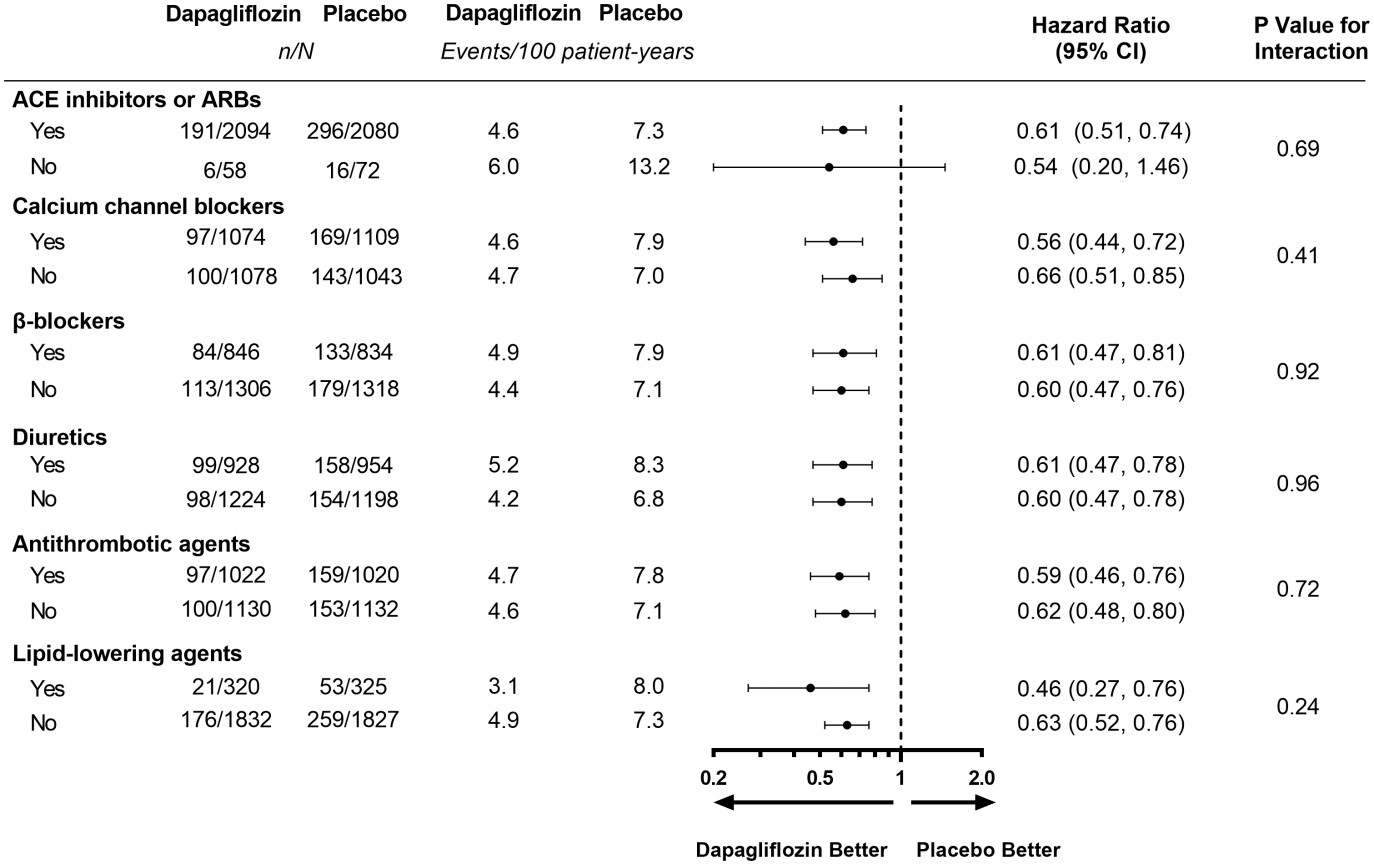
Primary composite end point by cardiovascular medication use/nonuse at baseline. Primary composite end point: ≥50% decline in eGFR, end‐stage kidney disease, or death from a kidney or cardiovascular cause. ACE indicates angiotensin‐converting enzyme; ARB, angiotensin receptor blocker; and eGFR, estimated glomerular filtration rate.

**Figure 2:**
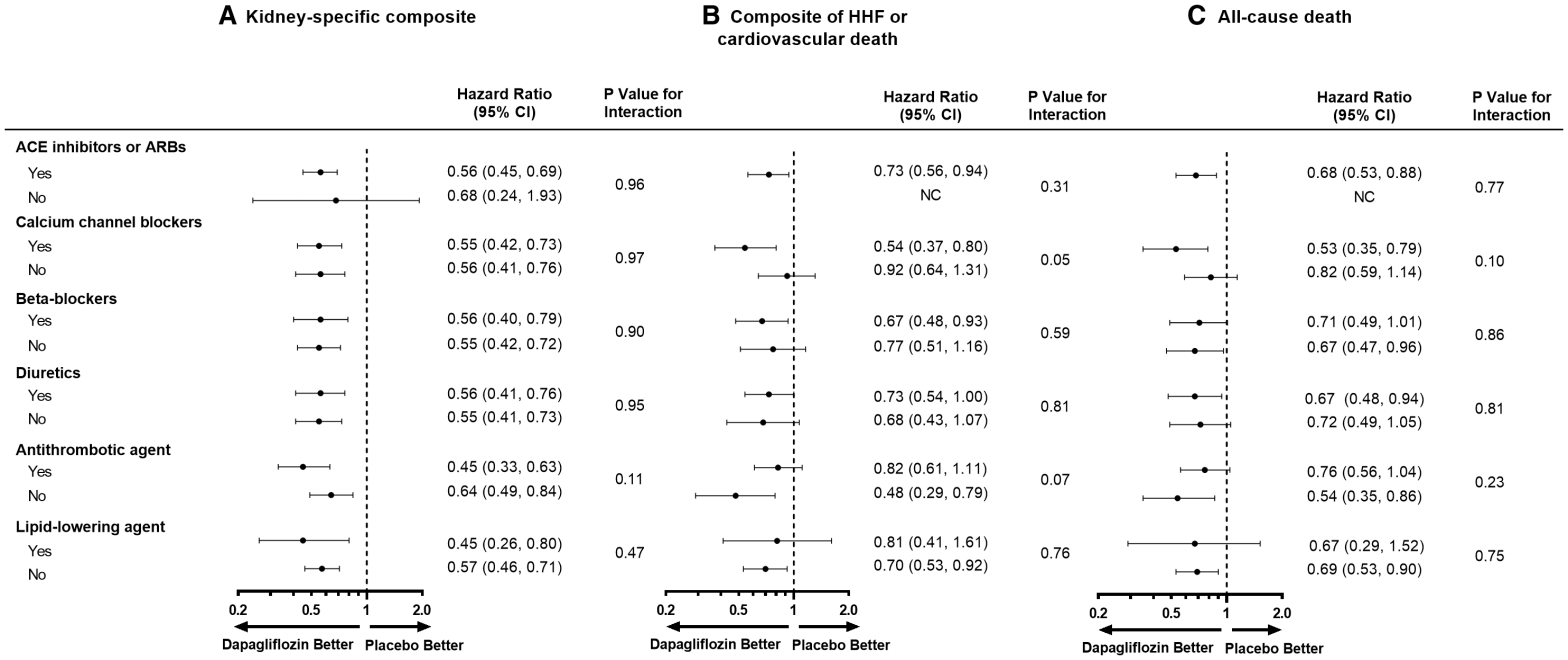
Secondary end points (A, kidney‐specific composite; B, composite of HHF or cardiovascular death; and C, all‐cause death) by cardiovascular medication use/nonuse at baseline. Kidney‐specific composite end point: ≥50% decline in eGFR, end‐stage kidney disease, or death from a kidney cause. ACE indicates angiotensin‐converting enzyme; ARB, angiotensin receptor blocker; eGFR, estimated glomerular filtration rate; and HHF, hospitalization for heart failure; NC, not calculable.

Figure 1 shows the effects of dapagliflozin on the primary composite end point in randomized participants by use/nonuse of cardiovascular medications. Figure 2A through 2C show corresponding effects of dapagliflozin on the secondary efficacy end points. While there were slight differences in the point estimates for the relative and absolute risk reductions across use/nonuse of specific cardiovascular medications, there was no clear evidence of heterogeneity. In other words, there was no evidence that the beneficial effects of dapagliflozin were enhanced or diminished by any of the cardiovascular medications examined.

### Relative Efficacy of Dapagliflozin by Baseline Dose of RAAS Inhibitors

**Figure 3:**
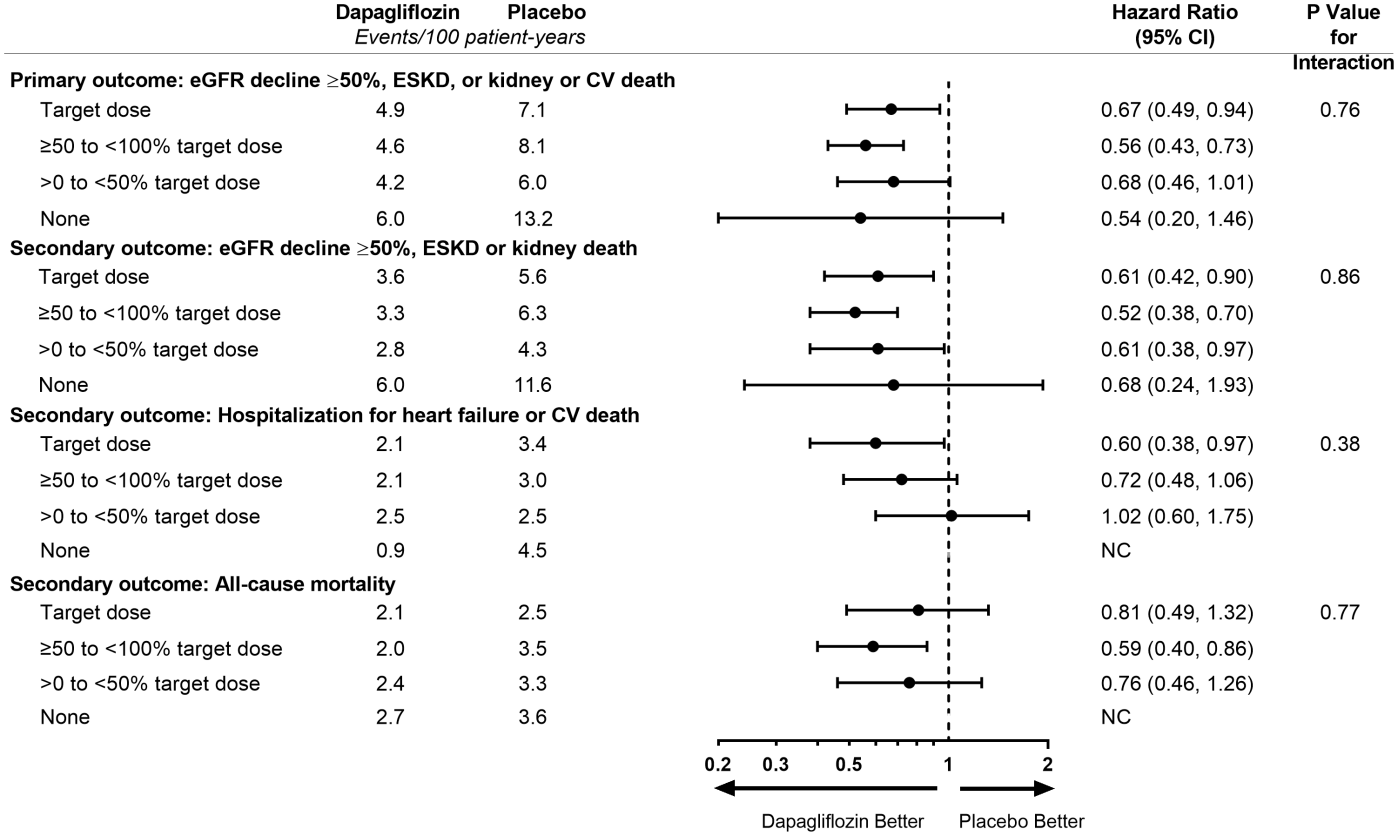
Primary and secondary end points by baseline ARB or ACEi dose. ACEi indicates angiotensin‐converting enzyme inhibitor; ARB, angiotensin receptor blocker; CV, cardiovascular; eGFR, estimated glomerular filtration rate; ESKD, end‐stage kidney disease; and NC, not calculable.

Among patients randomized to placebo, event rates for the primary composite end point were highest among the 3.0% of participants untreated with RAAS inhibitors (13.2 events per 100 patient‐years); event rates were similar among the 3 other RAAS inhibitor dose groups (7.1, 8.1, and 6.0 events per 100 patient‐years for 100% [or more], 50 to <100%, and <50% of the maximum labeled antihypertensive dose, respectively). Dapagliflozin compared with placebo consistently reduced the risk of the primary and secondary end points irrespective of the RAAS inhibitor dose group (Figure 3).

The effects of dapagliflozin (versus placebo) on attenuating the decline in eGFR was evident irrespective of the dose of the prescribed RAAS inhibitor dose (Figure S1, interaction *P*=0.88).

### Safety of Dapagliflozin by Baseline Use/Nonuse of Cardiovascular Medications

Table 2 shows a summary of serious adverse events and adverse events leading to discontinuation in patients stratified by baseline use/nonuse of cardiovascular medications, with safety outcomes by RAAS inhibitor dose in Table S1. We were particularly interested in whether concomitant use of dapagliflozin and diuretic agents (used in the treatment of heart failure or hypertension) would increase the risk of hypotension, acute kidney injury, falls, or other complications, owing to the natriuretic effects of dapagliflozin. At baseline, 1056 (24.6%) patients were treated with loop diuretics, and 906 (21.1%) were treated with thiazide diuretics. We did not observe an increased risk of serious adverse events with concomitant dapagliflozin and diuretic use. While the proportion of patients treated with diuretics at baseline who experienced an adverse event leading to discontinuation of study drug was numerically higher among patients randomized to dapagliflozin compared with those randomized to placebo, the favorable effects of dapagliflozin on the primary composite end point were similar among patients treated and not treated with loop diuretics (interaction *P*=0.86) and those treated and not treated with thiazide diuretics (interaction *P*=0.46).

**Table 2:** SAEs and AEsLeading to Discontinuation in Patients Stratified by Baseline Use/Nonuse of Cardiovascular Medications

AE, n/N (%)	With use of cardiovascular medication		Without use of cardiovascular medication	
	Dapagliflozin	Placebo	Dapagliflozin	Placebo
ACEi use				
Any AE leading to discontinuation of the study drug	39/673 (5.8)	30/678 (4.4)	79/1476 (5.4)	93/1471 (6.3)
Any SAE*	211/673 (31.4)	229/678 (33.8)	422/1476 (28.6)	500/1471 (34.0)
ARB use				
Any AE leading to discontinuation of the study drug	76/1441 (5.3)	86/1426 (6.0)	42/708 (5.9)	37/723 (5.1)
Any SAE*	406/1441 (28.2)	482/1426 (33.8)	227/708 (32.1)	247/723 (34.2)
Calcium channel blocker use				
Any AE leading to discontinuation of the study drug	56/1073 (5.2)	67/1108 (6.0)	62/1076 (5.8)	56/1041 (5.4)
Any SAE*	311/1073 (29.0)	412/1108 (37.2)	322/1076 (29.9)	317/1041 (30.5)
β‐blocker use				
Any AE leading to discontinuation of the study drug	44/843 (5.2)	46/833 (5.5)	74/1306 (5.7)	77/1316 (5.9)
Any SAE*	292/843 (34.6)	345/833 (41.4)	341/1306 (26.1)	384/1316 (29.2)
Diuretic use				
Any AE leading to discontinuation of the study drug	56/927 (6.0)	57/953 (6.0)	62/1222 (5.1)	66/1196 (5.5)
Any SAE*	330/927 (35.6)	391/953 (41.0)	303/1222 (24.8)	338/1196 (28.3)
Antithrombotic agent use				
Any AE leading to discontinuation of the study drug	52/1022 (5.1)	62/1018 (6.1)	66/1127 (5.9)	61/1131 (5.4)
Any SAE*	368/1022 (36.0)	414/1018 (40.7)	265/1127 (23.5)	315/1131 (27.9)
Lipid‐lowering agent use				
Any AE leading to discontinuation of the study drug	18/320 (5.6)	25/325 (7.7)	100/1829 (5.5)	98/1824 (5.4)
Any SAE*	99/320 (30.9)	120/325 (36.9)	534/1829 (29.2)	609/1824 (33.4)

ACEi indicates angiotensin‐converting enzyme inhibitor; AE, adverse event; ARB, angiotensin receptor blockers; and SAE, serious adverse event.

* Includes death.

## Discussion

We conducted this prespecified analysis to determine whether a variety of commonly prescribed cardiovascular medications modified the safety and efficacy of dapagliflozin in patients with CKD, with and without type 2 diabetes, a sizeable fraction of whom have underlying cardiovascular disease, including coronary, cerebral and peripheral arterial disease, heart failure, atrial fibrillation, and hypercholesterolemia. We found consistent benefits of dapagliflozin on the primary composite end point, as well as on the secondary end points, among patients treated and not treated with diuretics (loop diuretic, thiazide diuretic, or mineralocorticoid receptor antagonists), calcium channel blockers, β‐adrenergic antagonists, antithrombotic agents (antiplatelet agents or anticoagulants), and lipid‐lowering medications. Moreover, we found no evidence that use of any of these medication classes modified the safety profile of dapagliflozin.

In addition to examining whether cardiovascular medications modified the effects of dapagliflozin in this population, we took the opportunity to examine the effects of dapagliflozin in patients based on baseline dosing of ACE inhibitors or ARBs. While higher doses of ACE inhibitors or ARBs are generally thought to afford patients a greater benefit than lower doses,^6^ dosing in practice is frequently limited by hypotension or hyperkalemia, and some patients are intolerant of troublesome side effects (eg, cough) or serious allergic reactions (eg, angioedema) seen more frequently with ACE inhibitors. We found consistent relative and absolute benefits of dapagliflozin across the range of RAAS inhibitor doses.

Our results generally confirm findings observed in other populations treated with dapagliflozin and in patients with diabetic kidney disease treated with dapagliflozin and other SGLT2 inhibitors. In the DAPA‐HF trial, in which the primary composite end point was cardiovascular death or worsening heart failure, Docherty et al found consistent benefits of dapagliflozin by use/nonuse or dose of a variety of background heart failure treatments, including diuretics, RAAS inhibitors, and β‐adrenergic antagonists in patients with heart failure with reduced ejection fraction.^7^ Verma et al found similar results in the EMPEROR‐Reduced trial in which patients were randomized to empagliflozin or placebo^8^; both studies also examined effects of SGLT2 inhibitors with and without background use of guideline‐concordant therapies in combination. These analyses did not examine effects of background heart failure therapies on incidence or progression of kidney disease. In an analysis of data from the EMPA‐REG OUTCOME trial, Mayer et al showed that the effects of empagliflozin on a classic kidney composite end point (ie, doubling of serum creatinine, initiation of dialysis, or death due to kidney disease) were consistent among patients irrespective of baseline use/nonuse of diuretics, calcium channel blockers, and RAAS inhibitors.^9^ The analyses we present here confirm and extend previously published findings by documenting the safety and efficacy of dapagliflozin with use/nonuse of a wide range of cardiovascular medications, demonstrating benefits on kidney end points among patients with and without type 2 diabetes, and among patients with mild, moderate, and advanced CKD, populations at higher risk of adverse drug effects relative to persons with normal or near normal kidney function.

While the effects of antithrombotic and lipid‐lowering agents on kidney function are not as profound as those of other cardiovascular drugs that alter renal hemodynamics, including diuretics, RAAS inhibitors, β‐adrenergic antagonists, and calcium channel blockers, antiplatelet agents, anticoagulants, statins, and other lipid‐lowering agents are commonly prescribed in patients with CKD, particularly those with type 2 diabetes, a history of cardiovascular events, and atrial fibrillation. The demonstrated safety of coadministration of dapagliflozin with a range of antithrombotic and lipid‐lowering agents should allow patients to enjoy the benefits of dapagliflozin on reducing risks of heart failure and progressive kidney disease, while safely deriving the benefits of treatment with other agents aimed to reduce complications of atherosclerotic vascular disease and to prevent ischemic and cardioembolic stroke.

RAAS inhibitors were initially shown to slow the progression of CKD more than 2 decades before publication of the CREDENCE and DAPA‐CKD trial results.^10^, ^11^, ^12^, ^13^ It is essential that we understand how best to deliver RAAS inhibitors and SGLT2 inhibitors in combination, particularly among patients with moderate‐to‐advanced CKD who experience relatively high rates of hyperkalemia. Dapagliflozin was efficacious irrespective of the level of the participants' maximally tolerated RAAS inhibitor dose, when the latter was described as the proportion of the maximal daily antihypertensive dose as outlined in the respective product label. Nearly three‐quarters of participants were treated with an ACE inhibitor or ARB at doses 50% or more of the maximal daily antihypertensive dose, ensuring that placebo‐treated patients were receiving guideline‐concordant care for CKD with albuminuria. A similar distribution of dosing of background ACE inhibitor or ARB therapy was recently reported from the FIDELIO‐DKD trial.^14^

While the bulk of the analyses presented here address efficacy end points, the safety findings are equally if not more meaningful. Despite theoretical risks associated with coadministration of SGLT2 inhibitors and diuretics, RAAS inhibitors, β‐adrenergic antagonists, and calcium channel blockers, we saw no increase in serious adverse events or adverse events of special interest when these agents were used in combination with dapagliflozin, or when several of these agents were used in combination. Indeed, serious adverse events were less frequent among patients treated with dapagliflozin compared with placebo.

There are several limitations to these analyses. First, while we can definitively state that there was no evidence of heterogeneity of the dapagliflozin effect depending on use/nonuse of cardiovascular medications or in the case of RAAS inhibitors, the proportion of maximum labeled antihypertensive dose at baseline, the power to detect interactions was limited, and we may have missed subtle but clinically meaningful accentuation or attenuation of effects that might be evident in larger patient samples. It may be informative to repeat these analyses using population‐based (“real world”) samples in future years. Second, given differences in practice patterns among and within geographic regions, we were unable to examine effect modification by individual agents, as the modest sample size of the trial required us to explore these interactions by drug class. Finally, although the broad inclusion criteria for the DAPA‐CKD trial resulted in inclusion of participants with many underlying causes of CKD, we had insufficient power to determine whether any cardiovascular drug class or individual agent exerted unique modifying effects in patients with immunoglobulin A nephropathy, focal segmental glomerulosclerosis, membranous nephropathy, or other specific causes of CKD.

## Conclusions

In summary, the safety profile and efficacy of dapagliflozin on kidney and cardiovascular end points in patients with CKD were consistent among patients treated and not treated with a range of cardiovascular medications.

## Sources of Funding

This study was funded by AstraZeneca. The funder of the study was involved in the study design, data analysis, data interpretation, writing of the report, and the decision to submit the paper for publication.

## Disclosures

Dr Chertow has received fees from AstraZeneca for service on the DAPA‐CKD trial steering committee. He serves on the Board of Directors for Satellite Healthcare. He has served on other trial steering committees for Akebia, AstraZeneca, Gilead, Sanifit, and Vertex, and on data safety monitoring boards for Bayer, Mineralys, and ReCor. He has served as an advisor and received fees and/or stock options from Ardelyx, CloudCath, Durect, Miromatrix, Outset, and Unicycive. He has received research grants from the National Institute of Diabetes and Digestive and Kidney Diseases, National Heart, Lung, and Blood Institute, and National Institute of Allergy and Infectious Diseases. Dr Correa‐Rotter has received fees from AbbVie, AstraZeneca, GlaxoSmithKline, Medtronic, and Boehringer Ingelheim, and has lectured for Amgen, Janssen, Takeda, AstraZeneca, and Boehringer Ingelheim and has received research support from GlaxoSmithKline, Novo Nordisk, and AstraZeneca. P. Vart reports receiving a travel grant from AstraZeneca. Dr McMurray has received payments to his employer, Glasgow University, for his work on clinical trials, consulting, and other activities from AstraZeneca, Cytokinetics, KBP Biosciences, Amgen, Bayer, Theracos, Ionis Pharmaceuticals, Dalcor Pharmaceuticals, Novartis, GlaxoSmithKline, Bristol Myers Squibb, Boehringer Ingelheim, Cardurion, and Alnylam, and has received personal lecture fees from Abbott, Alkem Metabolics, Eris Life Sciences, Hickma, Lupin, Sun Pharmaceuticals, Medscape/Heart.org, ProAdWise Communications, Radcliffe Cardiology, Servier, and the Corpus. Dr Rossing has received fees to Steno Diabetes Center Copenhagen for: steering group membership and/or lectures and advice from AstraZeneca, Novo Nordisk, Bayer, and Eli Lilly; advisory board participation from Sanofi Aventis and Boehringer Ingelheim; and steering group participation from Gilead. Dr Langkilde and Dr Sjöström are employees and stockholders of AstraZeneca. Dr Toto is a member of the Executive Committee of the DAPA‐CKD study, has received consulting fees from Boehringer Ingelheim, Reata Pharma, and Chinook, and payment from Medscape and Medical Education Resources, participated in advisory boards for Bayer and Vioforand on data monitoring committees for Akebia and Otsuka.

Dr Wheeler has received consultancy fees from AstraZeneca and personal fees from Amgen, Astellas, Bayer, Boehringer Ingelheim, Gilead, GlaxoSmithKline, Janssen, Napp, Mundipharma, Reata, Tricida, Vifor Fresenius, Merck Sharp and Dohme, ProKidney, Galderma, and Zydus. H.J.L. Heerspink has received honoraria (paid to his institution [University Medical Center Groningen]) for participation in steering committees from AstraZeneca, Janssen, Gilead, Bayer, Chinook, and CSL Pharma; honoraria for participation in advisory boards from Merck, Mitsubishi Tanabe, Janssen, and Mundipharma; fees for consultancy from AstraZeneca, AbbVie, Retrophin, Boehringer Ingelheim, and Novo Nordisk; and research grant support from AstraZeneca, AbbVie, Janssen, and Boehringer Ingelheim. The remaining authors have no disclosures to report.

## Supporting information


Data S1:
